# Sealing efficacy of titanium-doped versus conventional MTA in access perforation repair: An *in vitro* study

**DOI:** 10.6026/973206300212481

**Published:** 2025-08-31

**Authors:** Sneha A. Patil, Shamurailatpam Priyadarshini, Rajeswari Putchala, Vankala Gautam, Smita Durga Dutta, Heena Dixit Tiwari, Rahul Tiwari

**Affiliations:** 1 Department of Conservative Dentistry and Endodontics, KLE VK Institute of dental sciences, KLE Academy of Higher Education and Research, Belagavi, Karnataka, India; 2 Department of Conservative Dentistry and Endodontics, Dental College, RIMS, Imphal, Manipur, India; 3 Department of Conservative & Endodontics, ST. Joseph's Dental College, Duggirala, NTR University of health sciences andhra Pradesh, India; 4 Department of Conservative Dental Sciences and Endodontics, College of Dentistry, Qassim University, Kingdom of Saudi Arabia; 5 Commisionerate of Health and Family Welfare, Government of Telangana, Hyderabad, India; 6 Department of Oral and Maxillofacial Surgery, Narsinhbhai Patel Dental College and Hospital, Sankalchand Patel University, Visnagar, Gujarat, India

**Keywords:** Mineral trioxide aggregate (MTA), titanium-doped MTA, access perforation, microleakage, endodontic repair

## Abstract

Mineral trioxide aggregate (MTA) is known for good sealing and also for its biocompatibility. However, limitations such as extended setting time and potential leakage persist. Therefore, it is of interest to compare the sealing efficacy of titanium-doped MTA and conventional MTA in the repair of access perforations using *in vitro* dye penetration model fourty extracted human mandibular premolars were used. Titanium-doped MTA showed significantly less dye penetration (mean 0.92 mm) compared to conventional MTA (mean 1.46 mm) (p = 0.001), indicating superior sealing efficacy. Thus, Titanium-doped MTA demonstrates better sealing capability than conventional MTA and may serve as an improved material for perforation repair.

## Background:

Access perforations during endodontic procedures remain a significant clinical challenge, as they compromise the structural integrity of the tooth and can lead to microbial contamination, ultimately affecting the prognosis of the treatment. Successful management of perforations depends largely on the choice of repair material, which must provide an effective seal, be biocompatible and withstand functional stresses [1]. Among various biomaterials developed for this purpose, MTA has gained wide acceptance due to its superior sealing ability, biocompatibility and regenerative potential [2- 3]. Despite its favorable properties, conventional MTA has several drawbacks, including long setting time, difficult handling, potential for discoloration and questionable mechanical resistance under load [4- 5]. To overcome these limitations, modifications to MTA formulations have been explored. One such promising advancement is titanium-doped MTA, which involves the incorporation of titanium oxide nanoparticles into the MTA matrix [6]. Titanium compounds are known to enhance physical and biological properties due to their chemical stability, high biocompatibility and ability to promote bio mineralization [7]. *in vitro* studies have suggested that doping MTA with titanium can improve its compressive strength, accelerate setting time and increase resistance to microleakage [8]. This modification also aims to reduce the incidence of material washout and improve sealing ability in the presence of moisture-an essential requirement in perforation repair scenarios [9]. Given that microleakage at the perforation site is a critical determinant of long-term clinical success, evaluating the sealing efficacy of these materials becomes imperative [10]. Therefore, it is of interest to comparatively assess the sealing ability of titanium-doped MTA versus conventional MTA when used in repairing simulated access perforations in extracted human teeth.

## Methodology:

## Research design:

This *in vitro* experiment research was conducted to compare the sealing efficacy of "titanium-doped mineral trioxide aggregate (Ti-MTA)" and "conventional mineral trioxide aggregate (C-MTA)" in access perforation repair. Forty freshly extracted human single-rooted mandibular premolars were considered for the research based on standardized inclusion and exclusion criteria. Teeth with cracks, caries, restorations, or resorptive defects were excluded.

## Specimen preparation:

All teeth crowns were removed at the "cementoenamel junction CEJ" to obtain a standardized root length of 15mm. Root canals were prepared using ProTaper rotary files up to size F3 and irrigated with 2.5% sodium hypochlorite followed by 17% EDTA to clear the smear layer. Canals were dried with paper-point and access perforations were made in the coronal third of the buccal root surface using a round bur (ISO size 0.8 mm) under water coolant to simulate clinical iatrogenic perforations.

## Grouping and repair material placement:

The 40 specimens were randomly divided into 2 equal sets (n=20):

[1] Group A (Control): Access perforations repaired with conventional MTA

[2] Group B (Experimental): Access perforations repaired with titanium-doped MTA

Both materials were mixed as per manufacturer instructions and placed into the perforation site with a microcarrier. A wet cotton pellet was placed in the canal and all specimens were stored at 37°C in 100% humidity for 48 hours to ensure ample setting.

## Dye penetration and evaluation:

After setting, the external root surfaces were coated with two layers of nail varnish but around the perforation site. The samples were submerged in 2% methylene blue dye for 48 hours, then rinsed and sectioned longitudinally. Dye penetration was assessed under a stereomicroscope at 20x magnification. Linear dye penetration (in mm) from the external surface toward the canal space was measured using Image J software.

## Statistical analysis:

Mean dye penetration values were compared between sets using the independent t-test. A p-value of <0.05 was considered statistically significant using SPSS version 25.0.

## Results:

The mean dye penetration depth in Group A (Conventional MTA) was greater compared to Group B (Titanium-Doped MTA), indicating superior sealing ability in the latter. The variance between the two sets was statistically significant. Table 1 (see PDF) presents the mean and standard deviation of dye penetration values in millimeters for both groups. Titanium-doped MTA exhibited a mean leakage of 0.92 ± 0.31 mm, while conventional MTA showed higher leakage at 1.46 ± 0.42 mm. In addition, the frequency distribution of specimens by leakage severity (mild: <1 mm, moderate: 1-2 mm, severe: >2 mm) revealed that 75% of specimens in the titanium-MTA group had mild leakage, while only 30% of the conventional MTA group achieved the same. This pattern further supports the improved sealing efficacy of titanium-doped MTA (Table 2 - see PDF). These results demonstrate that titanium doping in MTA significantly enhances sealing capacity in access perforation repair, likely due to improved material adaptation and reduced porosity.

## Discussion:

The management of access perforations is a critical aspect of endodontic therapy and the material used for repair plays a significant role in determining the clinical outcome. This research aimed to compare the sealing efficacy of titanium-doped MTA with that of conventional MTA, using dye penetration as an indicator of microleakage. The results demonstrated that titanium-doped MTA showed significantly reduced dye penetration compared to conventional MTA, indicating superior sealing ability. The enhanced performance of titanium-doped MTA can be attributed to several material properties. Titanium oxide, when incorporated into MTA, is known to improve the crystalline structure, leading to reduced porosity and better marginal adaptation. This contributes to decreased pathways for microleakage and improves the integrity of the seal in moist environments, which is crucial in clinical scenarios involving perforations [11]. Moreover, titanium-doped formulations are reported to have faster setting times and improved handling characteristics, both of which can influence the clinical success of perforation repair [12]. Previous studies evaluating modifications of MTA with different nanoparticles have demonstrated similar improvements in sealing efficacy and physical properties. The biocompatibility of titanium-containing compounds also makes them suitable for endodontic applications, as they support favorable tissue responses without inducing inflammation or cytotoxic effects [13]. This is essential for achieving long-term healing and maintaining the integrity of periradicular tissues. Additionally, the reduction in severe leakage in the titanium-MTA group in this research suggests better adaptation and material stability over time. The absence of specimens with dye penetration greater than 2 mm in the titanium-MTA group may imply a more predictable sealing outcome, which is particularly valuable in perforation management cases that are otherwise at higher risk for failure [14]. It is important to note that while *in vitro* studies like this one provide controlled comparisons, they may not fully replicate clinical conditions such as blood contamination, functional stresses and host immune responses. Therefore, further *in vivo* studies and clinical trials are necessary to confirm these findings and establish long-term outcomes [15, 16, 17, 18, 19-20].

## Conclusion:

Titanium-doped MTA exhibited significantly better sealing efficacy than conventional MTA in access perforation repair. Its reduced microleakage, likely due to enhanced physical and structural properties, positions it as a promising material for clinical use. Further research is needed to validate these results in clinical scenarios and explore long-term biocompatibility and success rates.
